# The Impact of the First Wave of the COVID-19 Pandemic on Providing Special Care Dentistry: A Survey for Dentists

**DOI:** 10.3390/ijerph18062970

**Published:** 2021-03-14

**Authors:** Jacobo Limeres Posse, Maria T. van Harten, Caoimhin Mac Giolla Phadraig, Márcio Diniz Freitas, Denise Faulks, Alison Dougall, Blánaid Daly, Pedro Diz Dios

**Affiliations:** 1 Medical-Surgical Dentistry Research Group (OMEQUI), Health Research Institute of Santiago de Compostela (IDIS), University of Santiago de Compostela (USC), 15782 Santiago de Compostela, Spain; jacobo.limeres@usc.es (J.L.P.); pedro.diz@usc.es (P.D.D.); 2 School of Dental Sciences, Trinity College Dublin & Dublin Dental University Hospital, Dublin D02 F859, Ireland; maria.vanharten@dental.tcd.ie (M.T.v.H.); caoimhin.macgiollaphadraig@dental.tcd.ie (C.M.G.P.); alison.dougall@dental.tcd.ie (A.D.); Blanaid.Daly@dental.tcd.ie (B.D.); 3 Service d’Odontologie CROC, Université Clermont Auvergne & CHU Clermont-Ferrand, 63000 Clermont-Ferrand, France; denise.faulks@uca.fr

**Keywords:** COVID-19, dentistry, disability, special care dentistry, dental care

## Abstract

This study aimed to investigate the impact of COVID-19 on the experiences of special care dentistry providers worldwide. An online survey was administered from 10 to 31 July 2020. Age, sex, years of professional activity, COVID-19 status, geographical area of origin and length of lockdown period were recorded for all participating dentists. The relationships between these variables and the changes in clinical activity, the treated patients’ COVID-19 status and the implementation of protective measures in the dental clinic were analyzed. A total of 436 (70.6% women) dentists from 59 countries responded to the survey. Clinical activity was reduced or stopped for 79.1% of respondents. The most common change was to limit treatment to urgent care only (53.7%). Treatment under general anesthesia or deep sedation was discontinued (51.0%) or reduced (35.8%) for the majority of respondents. Male dentists were more likely to maintain their clinical activity than female dentists (*p* < 0.001), and respondents from North America were more likely to do so than participants from other geographical regions (*p* < 0.001). Dentists from Latin America and the Caribbean were more likely to report treatment of confirmed cases of COVID-19 than those from Europe (*p* < 0.001). The implementation of protective measures in the dental office was determined by the survey participant’s sex, intensity of clinical activity and geographical area of origin. To conclude, the provision of special care dentistry was considerably reduced in response to the pandemic. Service maintenance was mainly related to the geographical area in which the surveyed dentists worked, further exacerbating pre-existing inequalities.

## 1. Introduction

On the 31 of December 2019, the Health Department of the Chinese province of Hubei reported the presence of a cluster of 27 cases of pneumonia in the city of Wuhan [1]. The etiological agent responsible for this outbreak was a new coronavirus (2019-nCoV) [2], which the International Committee on Taxonomy of Viruses labeled Severe Acute Respiratory Syndrome Coronavirus-2 (SARS-CoV-2). On the 11 of February 2020, the World Health Organization (WHO) officially termed this new disease Coronavirus disease 2019 (COVID-19) and only a month later declared the COVID-19 pandemic [3]. By the 9 of February 2021, the COVID-19 epidemic had spread to 255 countries and caused 106.1 million confirmed cases and over 2.3 million deaths, according to the WHO [4].

Healthcare workers (HCWs) comprised an especially vulnerable group to SARS-CoV-2 infection during the first stages of the pandemic, when appropriate protective measures (e.g., personal protective equipment, dental office procedures for receiving patients and environmental cleaning and disinfection) had still not been established [5]. A recently published study that collected information from 195 countries recorded 152,888 cases and 1413 deaths among HCWs up to the 8 of May 2020, which represents 3.9% of the patients with a confirmed COVID-19 diagnosis and 0.5% of all deaths due to this cause worldwide [6]. In this study [6], general practitioners and mental health nurses were the specialties at greatest risk of death, but only one dental nurse was among those who died. After detecting SARS-CoV-2 in saliva samples, however, it was suggested that dentists and dental hygienists faced a particularly high risk of contracting COVID-19 due to the close face-to-face contact that they maintain with their patients and due to the potential exposure to contaminated saliva droplets and aerosols generated during dental procedures [7,8,9]. This risk was confirmed in other countries in the epicenter of the initial COVID-19 pandemic, such as Italy, which had recorded the deaths of 13 dentists by the 8 of May 2020, which represented 7.5% of all physicians and dentists who died in that country [10].

Accordingly, numerous international organizations and professional bodies released specific clinical practice guidelines for reducing the risk of transmitting SARS-CoV-2 in the dental setting. Although their recommendations were heterogeneous, at that time, most agreed on limiting care to emergency procedures, performing patient triage, wearing class 2 filtering facepiece masks and avoiding aerosol-generating procedures [11,12].

The implementation of these safety protocols should be particularly rigorous in certain medically compromised individuals, such as transplant recipients, persons with cancer, immunodeficiencies, severe respiratory conditions and adults with chronic kidney disease, given that they are clinically considered extremely vulnerable to COVID-19 [13]. Adults with Down’s syndrome have recently been included in this report [13], and it has been suggested that individuals with intellectual and developmental disabilities have a higher prevalence of specific comorbidities associated with a poorer COVID-19 prognosis [14]. Paradoxically, some of these individuals live in institutions or regularly visit day centers with high prevalence of COVID-19 and have difficulties implementing hand washing, social distancing or wearing facemasks. They may also be more likely to require emergency dental care (given that they have more oral disease and traumatic dental injuries), to be uncooperative or even to reject any dental care in the conventional setting [15,16,17].

The consequences of this scenario following the first wave of the COVID-19 pandemic, with limited access to healthcare services, altered routine care, suspended general anesthesia sessions and untreated oral disease, are relevant to special care dentistry (SCD) and lead to the deterioration of medical and dental conditions [18].

This survey aimed to investigate the impact of COVID-19 on the dental care provision, practice modifications and protective measures among dentists who work with individuals with disabilities and disadvantages worldwide (including frail persons and those with long-term medical conditions).

## 2. Materials and Methods

This study was designed as an online, self-administered, cross-sectional, anonymized survey and was approved by the Research Ethics Committee of the University of Santiago de Compostela (Spain) (reference code USC-10/2020).

To develop the survey, a battery of preliminary questions was drafted, which was reviewed and agreed upon by a panel of 7 SCD experts and a statistician. The definitive survey consisted of 67 questions (Supplementary Material File S1) grouped into 4 sections: (1) demographics and professional activity (5 questions); (2) the COVID-19 pandemic and its repercussions for clinical activity (17 questions); (3) changes in practice over time related to COVID-19 restrictions (42 questions); and (4) expectations for the future (3 questions with open responses). The analysis reported here focused on the responses to the questions in Section 1 and Section 2. A number of these questions refer to clinical activity conducted during “lockdown”, defined as “the most restrictive period of confinement according to the government public health measurements in each country”. 

The survey, directed at dentists who provide SCD worldwide, was available in English on Google Forms between the 10 and 31 of July 2020. To recruit participants, opportunistic sampling was used and the survey was disseminated on social media and promoted through global and regional SCD networks. Snowballing, by sharing the survey URL with professional colleagues working in SCD, was encouraged.

Statistical analysis was performed using SPSS program v.26 (IBM Corp, Armonk, NY, USA). Demographic data are presented using numbers and percentages. To analyze the relationship between variables such as sex, age, years of professional activity, geographical area of origin and length of lockdown, on one hand, and changes in clinical activity, COVID-19 status of treated patients and implementation of protective measures in the dental clinic, Pearson’s chi-squared test with Bonferroni correction was applied. To facilitate the analysis, respondents were redistributed by age into 2 groups (50 years or younger and older than 50 years), by geographical region into 4 groups (Europe, Latin America and the Caribbean, North America and others) and by changes in clinical activity into 2 groups (maintained/increased and reduced/stopped).

## 3. Results

The total number of participants was 436, residing in 59 countries. Maximum participation was recorded in the United States (88 participants; 20.2%), the United Kingdom (64 participants; 14.7%) and Brazil (58 participants; 13.3%). In contrast, 21 countries had only token representation with only one participant (Bangladesh, Christmas Island, Dominican Republic, Ecuador, Iraq, Isle of Man, Israel, Italy, Libya, Macedonia, Malta, Morocco, New Caledonia, Oman, Paraguay, Peru, Poland, Serbia, South Africa, Sweden and Uruguay). When analyzing the participation by geographical area (Table 1), the largest percentage of responses was obtained from European dentists (40.4%) and the smallest from African dentists (0.7%).

Of the respondents, 70.6% were women (*n* = 308), 29.1% were men (*n* = 127) and one of the participants declared a non-binary gender. Some 55.5% (*n* = 242) of the respondents were in the 30–50-year age range, 38.1% were older than 50 years (*n* = 166), and only 8.4% were younger than 30 years (*n* = 28).

More than half of the respondents had over 20 years of professional experience (*n* = 230; 52.8%), 28.2% (*n* = 123) had 10–20 years experience, and the remaining 19.0% (*n* = 83) had less than 10 years. Some 45.9% (*n* = 195) worked exclusively in the public sector, 29.6% (*n* = 126) worked in the private sector, and 24.5% (*n* = 104) worked in both.

Overall, 79.8% of the respondents lived in countries where the lockdown period lasted longer than 6 weeks (in 53.4%, the period lasted longer than 8 weeks). When analyzing the duration of the lockdown period according to geographical area, we found that Europe typically had the longest lockdowns (43.8% of all lockdowns longer than 8 weeks and 53.9% of those lasting 6–8 weeks). Conversely, 63.4% of the responders who did not undergo any lockdown and 36.2% of those whose lockdown period was shorter than 6 weeks lived in Latin America and the Caribbean (Table 2).

In July 2020, when the survey was disseminated, 72.7% (*n* = 317) of the participants stated that their country’s lockdown restrictions were “easing”, and 8.3% (*n* = 36) stated that the “restrictions had ended”. Only 5.3% (*n* = 23) were “currently in full lockdown”, and the remaining 13.8% (*n* = 60) “had not entered full lockdown”. 

In terms of the participants’ COVID-19 status (Table 3), almost 1 of every 3 (*n* = 141; 32.3%) had been tested for SARS-CoV-2 and/or for SARS-CoV-2 antibody titers. A COVID-19 diagnosis had been confirmed in 11 cases (2.5%), and one participant had been hospitalized due to the severity of symptoms. The participants’ COVID-19 status was not influenced by their sex or age. The percentage of confirmed COVID-19 cases among dentists responding from Latin America and the Caribbean was 7.9%, significantly greater than that detected in the remaining geographical areas (*p* < 0.001).

More than half of respondents had had a member of their team self-isolate (*n* = 249; 57.1%), 1 of every 5 stated that a member of their team had had a confirmed COVID-19 diagnosis (*n* = 87; 20.0%), and 9 (2.1%) stated that a member of their team had been hospitalized. All of these results are detailed in Table 3. Overall, 58.5% of the responders had a confirmed case of COVID-19 among their social contacts: 168 (38.5%) in their work center/institution, 125 (28.7%) among their closest friends or neighbors and 40 (9.2%) in their immediate family.

Some 24.8% (*n* = 108) of respondents considered that persons with intellectual and developmental disabilities were more susceptible to contracting COVID-19 in the dental clinic than the general population, and 28.7% (*n* = 125) considered that the risk of infection for the dentist was higher when treating these patients than the general population.

The first wave of COVID-19 caused substantial changes in the clinical activity of SCD providers (Table 4). The most common change was reducing clinical activity to urgent care, using face-to-face and teletriage (*n* = 234; 53.7%). Only 18.1% of the respondents (*n* = 79) maintained their clinical activity. The respondents’ clinical activity appeared dependent on sex (*p* < 0.001); 34.6% (*n* = 44) of men maintained or increased their professional activity compared with 15.2% (*n* = 47) of women. Neither the participants’ age nor their years of professional experience were related to change in clinical activity (*p* = 0.072 and *p* = 0.223, respectively). Some 37.6% of North American respondents maintained or increased their clinical activity compared with 17% of European participants and 9.9% of those from Latin America and the Caribbean (*p* < 0.001).

The length of lockdown period and change in clinical activity were dependent variables (*p* < 0.001); in countries in which there was no lockdown, 29.3% (*n* = 12 out of 41) of responding dentists maintained or increased their clinical activity. When the lockdown period was shorter than 8 weeks, the percentage was 29.6% (*n* = 48 out of 162); however, this fell to 13.3% (*n* = 31 out of 233) among respondents in countries with lockdowns longer than 8 weeks.

In terms of the patients’ COVID-19 status, 11.0% of the respondents (*n* = 48) stated having treated a patient with a confirmed COVID-19 diagnosis, and 16.9% (*n* = 74) had treated a patient with a suspected diagnosis. This finding was not related to the respondents’ sex or age or to the intensity of the clinical activity (maintained/increased vs. reduced/stopped). However, the surveyed dentists from Latin America and the Caribbean were more likely to report treating confirmed cases than those from Europe (19.8% and 9.1%, respectively; *p* < 0.001).

Although 93.8% of respondents (*n* = 409) stated having provided some dental procedures during the period, 80.0% (*n* = 349) stated that they had had to change the type of treatment offered. Female dentists were less likely to undertake treatment than male dentists (*p* = 0.013). Overall, 123 respondents (28.2%) stated that, during this period, they had had to reduce the number of treatment sessions under deep sedation or general anesthesia, and 175 (40.1%) had had to completely discontinue this service. According to 74.8% (*n* = 326) of respondents, national guidelines required that dental treatment was restricted to urgent care, and 38.5% (*n* = 168) considered that these guidelines disproportionately disadvantaged persons with intellectual and developmental disabilities.

Regarding the implementation of protective measures against SARS-CoV-2 in the dental setting, the most common measure was to increase waiting times between patients to allow for cross-infection control measures. This measure was applied by 78.8% of respondents (*n* = 344); conversely, only 7.7% (*n* = 34) used an aerosol box shield. Implementation of protective measures in the dental office was associated with the dentist’s sex, the intensity of clinical activity and geographical area of origin. Male dentists were more likely to report using high-volume suction and rubber dam than female dentists (*p* = 0.005 and *p* = 0.014, respectively). Dentists who maintained or increased their activity were more likely to report using high-volume suction (*p* = 0.045), rubber dam (*p* = 0.016) and additional filters or air decontamination machines (*p* < 0.001). Dentists in North America were more likely to report using high-volume suction, changing the usual composition of any pre-procedural mouth rinses and using additional filters or air decontamination machines than those from other regions (*p* < 0.001, *p* < 0.001 and *p* < 0.001, respectively). Dentists in North America were, however, the least likely to report extending waiting times between patients, opening windows after aerosol-generating procedures and altering the use of air-conditioning than those from other regions (*p* < 0.001, *p* < 0.001 and *p* < 0.001, respectively) (Table 5).

## 4. Discussion

The main finding of this online survey of dentists completed in July 2020 was that SCD treatment services were reduced or stopped for 79% of respondents due to the COVID-19 pandemic. In addition, 86% of respondents reported a reduction in or closure of general anesthesia or deep sedation dental services. Likewise, in a questionnaire-based study to evaluate general dentists’ responses globally, most respondents (80%) also indicated that their workplaces were closed as a result of the COVID-19 outbreak [19].

Numerous guidelines have been published for providing dental care during the COVID-19 era [20]. Although most share common proposals, a number of the guidelines incorporate peculiarities directed at specific groups, such as immunocompromised individuals with cancer [21] and those with congenital bleeding disorders [22]. Many of these protocols are unrealistic, perhaps due to the lack of evidence-based research on their efficacy [20]. To date, no proposals have been developed that focus on the personalized and necessary adjustments required for persons with intellectual and developmental disabilities [16,17]. To the best of our knowledge, this is the first study that has explored the responses of dentists who regularly provide SCD worldwide in the face of the COVID-19 pandemic. The justification for this study is confirmed by the respondents themselves, given that 38.5% of them considered that the available guidelines disadvantaged thosewith intellectual and developmental disabilities.

The prevalence of COVID-19 among the responding dentists was high, apparently confirming the statement by some respondents that the risk of infection for practitioners is higher when treating persons with intellectual and developmental disabilities. However, this prevalence was particularly high among the responding dentists from Latin America and the Caribbean, while in Europe and North America, the prevalence was similar to that detected among all dentists, regardless of their specialty [23,24,25]. The rate of respondents infected by the virus probably reflects the regional differences in the prevalence of COVID-19, which could also be overestimated, given that the dentists infected with the virus or with declared symptoms of the infection might be more willing to participate in the survey [23]. As a consequence, it appears that the risk of infection for dentists, including those who treat persons with intellectual and developmental disabilities, is not comparable to that of other health professionals (such as those who work in intensive care units), provided that they adopt the basic protection recommendations [18].

Sex, geographical region and lockdown duration were found to be related to whether clinical activity was maintained or not during the period. Female dentists maintained their clinical activity less often than males in the current survey. The professional practice of female dentists is still subject to certain social–cultural patterns in which the responsibilities for family caretaking continue to fall disproportionately on women [26,27]. This might explain why clinical activity was particularly reduced when the lockdown period was extended and schools were shut. It may also explain why dentists from Latin America and the Caribbean more often interrupted their clinical activity, given that it is a more culturally traditional region, in which most dentists are currently women [28]. In Europe, where the lockdowns were longest, SCD is predominantly practiced by women in the public sector. European governments were amongst those who explicitly prohibited dental treatment in the first wave of the pandemic. In contrast, North America had a higher percentage of dentists who maintained their clinical activity, a result that was probably partly due to the fact that, in the United States, only 33.4% of dentists are women [29].

A considerable minority of respondents (11%) had performed dental procedures on patients with a confirmed diagnosis of COVID-19. In a survey among European experts in oral and maxillofacial surgery or oral surgery facing dental emergencies during the pandemic, two thirds of the respondents suggested treating COVID-19 positive patients during the pandemic only in university hospitals, while one third suggested treating them also in private offices [30]. It is possible that the number of persons with intellectual and developmental disabilities referred to reference centers and hospital dental departments increased with the inrush of the pandemic, especially when dealing with suspected COVID-19 infection. In addition, a number of countries centralized their dental services to address the general population (e.g., emergency dental centers) during lockdown [31]. Considering that 70.4% of the respondents conducted all or part of their professional activity in the public sector, it is likely that they were the recipients of most of the infected patients.

One of the most common findings was a reduction in the number of treatment sessions provided under deep sedation or general anesthesia, probably as a consequence of the scarcity of personnel and hospital resources, as well as of the cancellation of non-emergency medical and dental care [18]. To alleviate this situation, the promotion of non-pharmacological behavioral control techniques has been proposed [17], but their efficacy is limited in certain groups of persons with intellectual and developmental disabilities, particularly in emergency situations without previous adaptation. Intravenous sedation has also been suggested as a safe and cost-efficient option for providing SCD during the COVID-19 outbreak [32], but many countries require the presence of an anesthesiologist [17].

The implementation of personal protective equipment and office disinfection measures has been shown to minimize the risk of cross-infection in the dental setting, even in hospital and university dental services, where a larger number of infected patients are likely to be concentrated [33,34]. Although the available evidence has not shown a direct relationship between dental treatment and COVID-19 transmission [20], it has been estimated that the risk for a United States dental healthcare professional of becoming infected and dying from COVID-19 during the peak of the pandemic was 1:13,000, when applying these measures [35]. More SCD providers in the present series (51.3%) reported using basic cross-infection measures such as rubber dams whenever appropriate and possible than did general dentists from 30 different countries across the world responding to an online survey (16%) [35]. However, the adoption of these measures was mixed and was essentially influenced by the respondents’ sex and geographical area. Female dentists less often employed high-power suction and rubber dams than the male dentists, as expected if we consider that female dentists were less likely to undertake treatment than their male counterparts. The impact of sex diversity in dentistry is a multidimensional factor, inter-related with other variables such as generational changes [36], whose analysis is beyond the objectives of this study. The geographical differences are particularly marked when analyzing the responses of the North American participants, who less frequently increased waiting times between patients to allow for cross-infection control measurements and less frequently opened windows after aerosol-generating procedures. Conversely, this group most often used high-power suction and air decontamination machines. These findings are not exclusive to SCD providers and agree, for example, with the measures adopted in a number of private dental clinics in New York during and shortly after the first wave of the pandemic [37]. Likewise, in a multisite survey of general dentists’ perspectives, the level of comfort with preventive measures during the COVID-19 pandemic was low and also showed significant differences between regions [30]. A survey conducted among Brazilian dentists concluded that the reduction in the number of patients treated weekly during the pandemic was significantly greater in the public clinics than in the private clinics due to merely financial reasons [38], which could have affected the care of persons with intellectual and developmental disabilities.

This study has a number of potential limitations and its results should be interpreted with caution. Given that a protocol was not available at the time that this survey was developed, it might show some design flaws in relation to the development and refinement of the questionnaire (e.g., lack of a pilot test and semantic adjustment of questionnaire) [39]. The small sample size might affect the study’s statistical power. Although the sample size appears small, the number of dentists who regularly provided SCD is limited, and 436 respondents equals 7% of the members of the International Association for Disability and Oral Health, which is the largest scientific society for this specialty worldwide. This percentage falls into the range of 5–20% of dentists in a given area who must be invited to participate in this type of survey [39]. The survey duration was necessarily short given that the COVID-19 situation is dynamic and heterogeneous from one geographical region to the other. At the time that the survey was conducted, more than 80% of the respondents had already come out of full lockdown, so prolonging the survey to attract more respondents was deemed inappropriate. Almost half of the responding dentists were concentrated in three countries (United States, United Kingdom, Brazil); however, the participants’ redistribution by geographical area allowed us to analyze the importance of this variable at the global level. We did not record the respondents’ academic degrees and it has been suggested that good practice regarding the COVID-19 pandemic could be associated with qualifications [40]. The predominance of women among the responding dentists could have affected the meaning of certain responses but probably reflects the growing presence of female dentists worldwide and particularly in special care dentistry. Indeed, this female predominance among respondents has also been observed in other international surveys among general dentists on practice modifications to combat the COVID-19 outbreak [35].

It has been suggested that the COVID-19 pandemic could represent an opportunity for SCD providers, because it has helped to increase the visibility of primary, community and hospital services, to improve communication abilities through teleconsultation, to promote non-pharmacological behavioral control techniques and to implement new measures to prevent cross-infection [41]. The results of this survey, taking into account its limitations, are not conducive to over-optimism, however. The dentists surveyed here felt that guidelines did not protect vulnerable patient groups and the closure of general anesthesia and deep sedation services for dental care impacts disproportionately on persons with intellectual and developmental disabilities.

Worldwide, people with disabilities are subject to inequality in oral health, in terms of both the prevalence of disease and unmet healthcare needs [42]. Understanding the underlying factors that perpetuate the oral health equity gap is mandatory for developing effective interventions that achieve meaningful improvements in oral health for the most vulnerable [43]. Overall, the results of this survey are important in that they demonstrate that access to services was massively reduced for SCD patients during the early pandemic period, with reduced access to general anesthesia likely to strongly impact this patient group. In addition, a sizeable percentage of respondents felt that national guidelines had disproportionately disadvantaged persons with intellectual and developmental disabilities. The mistakes made in this initial emergency period of the pandemic must be redressed as the world moves into long-term strategies. Individuals with intellectual and developmental disabilities must be prioritized for care and SCD professionals must be prepared to advocate for their needs.

## 5. Conclusions

The dental care provided to persons with intellectual and developmental disabilities after the declaration of the pandemic has been heterogenous worldwide and was fundamentally determined by the geographical area where the surveyed dentists work. Although the differences between regions might have been affected by the chronology of regional outbreaks and the speed of the response to COVID-19, these aspects probably reflect social–economic and cultural differences and exacerbate the pre-existing inequalities in accessing specialist dental care.

## Figures and Tables

**Table 1:** Distribution of surveyed dentists by geographical area.

Geographical Area	Frequency	Percent
Africa *	3	0.7
Asia (except Near East) *	34	7.8
Europe	176	40.4
Latin America and the Caribbean	101	23.2
North America	93	21.3
Near East *	19	4.4
Oceania *	10	2.3
Total	436	100.0

* Grouped with “Others” (Africa + Asia + Near East + Oceania).

**Table 2:** Length of lockdown in terms of geographical area.

Lockdown Length (LDL)		Africa	Asia *	Europe	Latin America and the Caribbean	North America	Near East	Oceania	Total
>8 weeks	*N* dentists	1	22	102	49	48	6	5	233
	% within >8 week LDL	0.4	9.4	43.8	21.0	20.6	2.6	2.1	100.0
	% of Total	0.2	5.0	23.4	11.2	11.0	1.4	1.1	53.4
6–8 weeks	*N* dentists	0	3	62	9	30	7	4	115
	% within 6–8 week LDL	0.0	2.6	53.9	7.8	26.1	6.1	3.5	100.0
	% of Total	0.0	0.7	14.2	2.1	6.9	1.6	0.9	26.4
<6 weeks	*N* dentists	2	2	11	17	10	4	1	47
	% within <6 week LDL	4.3	4.3	23.4	36.2	21.3	8.5	2.1	100.0
	% of Total	0.5	0.5	2.5	3.9	2.3	0.9	0.2	10.8
No lockdown	*N* dentists	0	7	1	26	5	2	0	41
	% no lockdown	0.0	17.1	2.4	63.4	12.2	4.9	0.0	100.0
	% of Total	0.0	1.6	0.2	6.0	1.1	0.5	0.0	9.4
Total	*N* dentists	3	34	176	101	93	19	10	436
	% of Total	0.7	7.8	40.4	23.2	21.3	4.4	2.3	100.0

* Except Near East.

**Table 3:** History of COVID-19 infection amongst responding dentists (*n* = 436) and team members.

Participants’ COVID-19 Status	Responding Dentists		Other Dental Team Members	
	Responses	Percent of Cases	Responses	Percent of Cases
Quarantine/Self-isolation	129	29.6	249	57.1
Symptoms suggesting COVID-19	35	8.0	112	25.7
Tested for SARS-CoV-2 and/or for SARS-CoV-2 antibody titers	141	32.3	203	46.6
Confirmed COVID-19 diagnosis	11	2.5	87	20.0
Hospitalized due to COVID-19 symptoms	1	0.2	9	2.1
Not tested for COVID-19	254	58.3	75	17.2

**Table 4:** Changes in the clinical activity of the responding dentists during the initial wave of COVID-19 (*n* = 436).

Changes in Clinical Activity	Responding Dentists	
	Responses	Percent of Cases
Increased professional activity	12	2.8
Maintained professional activity	79	18.1
Reduced professional activity to urgent care, using face-to-face and teletriage	234	53.7
Reduced professional activity to urgent care, using teletriage only	62	14.2
Stopped all professional activity	49	11.2

**Table 5:** Implementation of protective measures against SARS-CoV-2 in the dental office, according to the responding dentists’ geographical area of origin (*n* = 436).

Protective Measures against SARS-CoV-2	Total	Europe	Latin America and the Caribbean	North America	Others	*p* *
	Responses (% of Cases)	Responses (% of Cases)	Responses (% of Cases)	Responses (% of Cases)	Responses (% of Cases)	
Increased the waiting time between patients to allow for cross-infection control measures	344 (78.8)	146 (83.0)	90 (89.1)	60 (64.5)	49 (74.2)	<0.001
Avoided generating aerosols whenever possible	343 (78.6)	137 (77.8)	84 (83.2)	65 (69.9)	58 (87.9)	0.030
Used high-power suction whenever possible	271 (62.1)	108 (61.4)	50 (49.5)	75 (80.6)	39 (59.1)	<0.001
Used rubber dam whenever appropriate and possible	122 (51.3)	86 (48.9)	44 (43.6)	51 (54.8)	43 (65.2)	0.039
Opened windows after aerosol-generating procedures	210 (48.1)	111 (63.1)	67 (66.3)	7 (7.5)	26 (39.4)	<0.001
Changed the usual composition of any pre-procedural mouth rinses	172 (39.4)	51 (29.0)	35 (34.7)	56 (60.2)	30 (45.5)	<0.001
Altered the use of air-conditioning in the dental surgery	122 (27.9)	58 (33.0)	39 (38.6)	14 (15.1)	11 (16.7)	<0.001
Used additional filters or air decontamination machines after aerosol-generating procedures	91 (20.8)	18 (10.2)	12 (11.9)	45 (48.4)	16 (24.2)	<0.001
Used an aerosol box shield	34 (7.7)	7 (4.0)	11 (10.9)	8 (8.6)	8 (12.1)	0.082

* Chi-squared statistic.

## Data Availability

Data sharing is not applicable to this article.

## Supplementary Materials

The following are available online at https://www.mdpi.com/1660-4601/18/6/2970/s1, File S1: Special Care Dentistry provision during the COVID-19 pandemic (a survey for dentists).
