# Supplementary material for: The Impact of the First Wave of the COVID-19 Pandemic on Providing Special Care Dentistry: A Survey for Dentists
Source: Int J Environ Res Public Health. 2021 Mar 14;18(6):2970. doi: 10.3390/ijerph18062970 (PMC8001730; doi:10.3390/ijerph18062970)
Supplement: Supplementary file 1 [file ijerph-18-02970-s001.pdf]

Special Care Dentistry provision during the COVID-19 pandemic (a survey for dentists)

*Required

SECTION 1. DEMOGRAPHIC / PROFESSIONAL INFORMATION

1. In which country do you practice dentistry? *

2. Gender *

Mark only one oval.

- Male
- Female
- Non binary
- Other

3. Age *

Mark only one oval.

- < 30 years
- 30-50 years
- > 50 years

4. How many years have you practised dentistry? *

Mark only one oval.

- < 10 years
- 10-20 years
- > 20 years

5. Which of the following best describes the health sector that you work in? *

Mark only one oval.

- Public
- Private
- Public & Private
- Voluntary organization such as charity / religious
- Other

SECTION 2. THE COVID-19 PANDEMIC

6. To the best of your knowledge, for how many weeks did the full coronavirus lockdown period last in your country? *

Mark only one oval.

- There was no lockdown
- < 2 weeks
- 2-4 weeks
- 4-6 weeks
- 6-8 weeks
- > 8 weeks

7. Which of the following best describes the status in your country today? *

Mark only one oval.

- ( ) In my country, we have not entered full lockdown
- ( ) In my country, we are currently in full lockdown
- ( ) In my country, lockdown restrictions are easing
- ( ) In my country, restrictions have ended

8. Your COVID-19 status. Tick all that apply *

Tick all that apply.

- [ ] I have been in quarantine / self-isolation
- [ ] I have had symptoms suggesting COVID-19
- [ ] I have been tested for SARS-CoV-2
- [ ] I have had a confirmed diagnosis of COVID-19
- [ ] I have been hospitalised because of COVID-19 symptoms
- [ ] I have been tested for SARS-CoV-2 antibody titers
- [ ] I was not tested for COVID-19

9. COVID-19 status of immediate members of your dental team. Tick all that apply

Tick all that apply.

- [ ] One or more have been in quarantine / self-isolation
- [ ] One or more have had symptoms suggesting COVID-19
- [ ] One or more have been tested for SARS-CoV-2
- [ ] One or more have had a confirmed diagnosis of COVID-19
- [ ] One or more have been hospitalised because of COVID-19 symptoms
- [ ] One or more have been tested for SARS-CoV-2 antibody titers
- [ ] None of the above

10. COVID-19 status of your social contacts. Tick all that apply *

Tick all that apply.

- [ ] There was at least one confirmed case in my work centre/institution
- [ ] There was at least one confirmed case in my immediate family
- [ ] There was at least one confirmed case in my closest friends or neighbours
- [ ] None of the above

11. Which of the following best describes the change to your clinical activity since COVID 19? *

Mark only one oval.

- ( ) I have stopped all professional activity
- ( ) I have reduced professional activity to urgent care, using tele-triage only
- ( ) I have reduced professional activity to urgent care, using face-to-face and tele-triage
- ( ) I have maintained my professional activity
- ( ) I have increased my professional activity

12. Which of the following best describes the impact of National Guidance in your country on the provision of dental care during the COVID-19 pandemic? *

Mark only one oval.

- ( ) There was no National Guidance in my country
- ( ) National Guidance meant that all dental procedures were prohibited
- ( ) National Guidance meant that dental care was restricted to urgent care
- ( ) National Guidance meant that dental care was restricted to non-aerosol generating procedures
- ( ) National Guidance meant that dental care was not restricted

13. Did you feel that your National Guidance disproportionately disadvantaged special care patients during the COVID-19 pandemic? *

Mark only one oval.

- Yes
- No
- Don’t know

14. If yes, briefly explain why?

____

15. Were you re-purposed (e.g., swabbing / tracing) or given alternative roles in the wider health and social care team? *

Mark only one oval.

- Yes
- No

16. If yes, what was this role?

____

17. Regarding your patients’ COVID-19 status: have you treated any COVID-19 patients from the start of the pandemic up to today...Tick all that apply *

Tick all that apply.

- [ ] Confirmed cases
- [ ] Suspected cases
- [ ] Recovered cases
- [ ] Don't know
- [ ] None of the above

18. On arrival in the dental setting...Tick all that apply *

Tick all that apply.

- [ ] Only patients with a recent COVID-19 test were accepted
- [ ] Only patients with a defined time elapsed since infection were accepted
- [ ] All patients had their temperature taken
- [ ] We asked specific questions aimed at detecting COVID-19 infection
- [ ] We did not triage patients based on COVID-19 status

19. Did the COVID-19 pandemic change the kind of dental procedures that you provide? *

Mark only one oval.

- Yes
- No

20. How did the COVID-19 pandemic impact on your ability to provide care under general anesthesia or sedation? *

Mark only one oval.

- The number of sessions increased
- The number of sessions decreased
- We were unable to provide care under general anesthesia or sedation
- There were no changes
- None of the above

21. How did the COVID-19 pandemic impact your dental setting and procedures...Tick all that apply *

Tick all that apply.

- [ ] I have avoided generating aerosols at all times
- [ ] I have avoided generating aerosols whenever possible
- [ ] I have used an aerosol box shield
- [ ] I have changed the usual composition of any pre-procedural mouthrinses
- [ ] I have used high power suction whenever possible
- [ ] I have used rubber dam whenever appropriate and possible
- [ ] I have opened windows after aerosol generating procedures
- [ ] I have used additional filters or air de-contamination machines (e.g. fogging) after aerosol generating procedures
- [ ] I have altered my use of air-conditioning in the dental surgery
- [ ] I have increased the waiting time between patients to allow for cross infection control measures
- [ ] I did not undertake any dental procedures

22. Do you think that your risk of contracting COVID-19 was higher when treating special care patients than when treating the general population? *

Mark only one oval.

- ( ) Yes
- ( ) No

23. Do you think that your special care patients were more likely to contract COVID-19 in the dental surgery than the general population? *

Mark only one oval.

- ( ) Yes
- ( ) No

24. Do you think that most of your special care patients were more likely to experience severe disease if they contracted COVID-19 than the general population? *

Mark only one oval.

- Yes
- No

SECTION 3. BEFORE the covid-19 pandemic

THE WEEK BEFORE COVID-19 RESTRICTIONS

25. What digital services did you use to communicate with patients?. Tick all that apply *

THE WEEK BEFORE COVID-19 RESTRICTIONS

Tick all that apply.

- [ ] Use of personal telephone
- [ ] Use of email
- [ ] Use of social messaging (Facebook, WhatsApp, Discord…)
- [ ] Use of non-dedicated videoconferencing (Skype, Zoom, Facetime…)
- [ ] Use of a dedicated teleconsultation system
- [ ] None of the above

26. What type of dentistry did you usually provide?. Tick all that apply *

THE WEEK BEFORE COVID-19 RESTRICTIONS

Tick all that apply.

- [ ] Special Care Dentistry
- [ ] General dentistry
- [ ] Paediatric dentistry
- [ ] Oral surgery
- [ ] Implantology
- [ ] Endodontics
- [ ] Periodontics
- [ ] Prosthodontics
- [ ] Orthodontics
- [ ] Oral Medicine
- [ ] Others

27. Did you usually have regular access to services under...Tick all that apply *

THE WEEK BEFORE COVID-19 RESTRICTIONS

Tick all that apply.

- [ ] Oral conscious sedation (e.g. diazepam)
- [ ] Relative analgesia/nitrous oxide conscious sedation
- [ ] Intravenous conscious sedation (e.g. midazolam)
- [ ] Intravenous deep sedation (e.g. propofol)
- [ ] General anaesthesia
- [ ] None of the above

28. Your patients with special needs were mostly... *

THE WEEK BEFORE COVID-19 RESTRICTIONS

Mark only one oval.

- ( ) Children
- ( ) Adults
- ( ) Elderly
- ( ) All ages

29. What categories of patients with special needs (principal diagnosis) did you usually provide care for? Tick all that apply *

THE WEEK BEFORE COVID-19 RESTRICTIONS

Tick all that apply.

- [ ] Physical disability
- [ ] Intellectual / Developmental disability
- [ ] Neurological disorders
- [ ] Sensory disability
- [ ] Medically compromised patients
- [ ] Frail older adults
- [ ] Mental Health / Socially excluded
- [ ] Anxious / Phobic patients

Other: [ ] ____________________

30. How many patients with special needs did you usually see in a week? *

THE WEEK BEFORE COVID-19 RESTRICTIONS

Mark only one oval.

- ( ) None
- ( ) 1-10 patients
- ( ) 11-20 patients
- ( ) 21-30 patients
- ( ) 31-40 patients
- ( ) > 40 patients

31. Did you offer domiciliary dental care? *

THE WEEK BEFORE COVID-19 RESTRICTIONS

Mark only one oval.

- ( ) Yes
- ( ) No

32. Did you find that patients were reluctant to attend for dental treatment due to fear of infection? *

THE WEEK BEFORE COVID-19 RESTRICTIONS

Mark only one oval.

- Yes
- No

33. Did you find that dental staff were reluctant to participate in the provision of dental treatment due to fear of infection? *

THE WEEK BEFORE COVID-19 RESTRICTIONS

Mark only one oval.

- Yes
- No

34. During oral exam and NON-aerosol generating dental procedures, in patients with unknown COVID-19 status, did you put on...Tick all that apply *

THE WEEK BEFORE COVID-19 RESTRICTIONS

Tick all that apply.

- [ ] A surgical mask
- [ ] A FFP2/FFP3/N95 respirator mask without a valve
- [ ] A FFP2/FFP3/N95 respirator mask with valve plus a surgical mask
- [ ] Goggles and/or a shield or visor
- [ ] A second pair gloves
- [ ] A surgical cap
- [ ] Disposable shoe coverings
- [ ] N/A

35. During aerosol generating dental procedures (AGPs), in patients with unknown COVID-19 status, did you put on...Tick all that apply *

THE WEEK BEFORE COVID-19 RESTRICTIONS

Tick all that apply.

- [ ] A surgical mask
- [ ] A FFP2/FFP3/N95 respirator mask without a valve
- [ ] A FFP2/FFP3/N95 respirator mask with valve
- [ ] Goggles and/or a shield or visor
- [ ] A second pair gloves
- [ ] A surgical cap
- [ ] Disposable shoe coverings
- [ ] I did not undertake any aerosol generating procedure
- [ ] N/A

36. Did all the clinical dental team members use similar levels of protective equipment? *

THE WEEK BEFORE COVID-19 RESTRICTIONS

Mark only one oval.

- ( ) Yes
- ( ) No
- ( ) N/A

37. . Did you have problems acquiring adequate personal protective equipment? *

THE WEEK BEFORE COVID-19 RESTRICTIONS

Mark only one oval.

- ( ) A lot
- ( ) Some
- ( ) A little
- ( ) None

38. How confident were you that you had sufficient information to have optimal infection prevention control at work? *

THE WEEK BEFORE COVID-19 RESTRICTIONS

Mark only one oval.

- Very confident
- Confident
- Not really confident
- Not confident at all

SECTION 4. DURING the covid-19 pandemic

DURING THE EMERGENCY FULL LOCKDOWN

39. What digital services did you use to communicate with patients?. Tick all that apply *

DURING THE EMERGENCY FULL LOCKDOWN

Tick all that apply.

- [ ] Use of personal telephone
- [ ] Use of email
- [ ] Use of social messaging (Facebook, WhatsApp, Discord...)
- [ ] Use of non-dedicated videoconferencing (Skype, Zoom, Facetime...)
- [ ] Use of a dedicated teleconsultation system
- [ ] None of the above

40. What type of dentistry did you usually provide?. Tick all that apply *

DURING THE EMERGENCY FULL LOCKDOWN

Tick all that apply.

- [ ] Special Care Dentistry
- [ ] General dentistry
- [ ] Paediatric dentistry
- [ ] Oral surgery
- [ ] Implantology
- [ ] Endodontics
- [ ] Periodontics
- [ ] Prosthodontics
- [ ] Orthodontics
- [ ] Oral Medicine
- [ ] Others

41. Did you usually have regular access to services under...Tick all that apply *

DURING THE EMERGENCY FULL LOCKDOWN

Tick all that apply.

- [ ] Oral conscious sedation (e.g. diazepam)
- [ ] Relative analgesia/nitrous oxide conscious sedation
- [ ] Intravenous conscious sedation (e.g. midazolam)
- [ ] Intravenous deep sedation (e.g. propofol)
- [ ] General anaesthesia
- [ ] None of the above

42. Your patients with special needs were mostly... *

DURING THE EMERGENCY FULL LOCKDOWN

Mark only one oval.

- ( ) Children
- ( ) Adults
- ( ) Elderly
- ( ) All ages

43. What categories of patients with special needs (principal diagnosis) did you usually provide care for? Tick all that apply *

DURING THE EMERGENCY FULL LOCKDOWN

Tick all that apply.

- [ ] Physical disability
- [ ] Intellectual / Developmental disability
- [ ] Neurological disorders
- [ ] Sensory disability
- [ ] Medically compromised patients
- [ ] Frail older adults
- [ ] Mental Health / Socially excluded
- [ ] Anxious / Phobic patients

Other: [ ] ____________________

44. How many patients with special needs did you usually see in a week? *

DURING THE EMERGENCY FULL LOCKDOWN

Mark only one oval.

- ( ) None
- ( ) 1-10 patients
- ( ) 11-20 patients
- ( ) 21-30 patients
- ( ) 31-40 patients
- ( ) > 40 patients

45. Did you offer domiciliary dental care? *

DURING THE EMERGENCY FULL LOCKDOWN

Mark only one oval.

- ( ) Yes
- ( ) No

46. Did you find that patients were reluctant to attend for dental treatment due to fear of infection? *

DURING THE EMERGENCY FULL LOCKDOWN

Mark only one oval.

- ◯ Yes
- ◯ No

47. Did you find that dental staff were reluctant to participate in the provision of dental treatment due to fear of infection? *

DURING THE EMERGENCY FULL LOCKDOWN

Mark only one oval.

- ◯ Yes
- ◯ No

48. During oral exam and NON-aerosol generating dental procedures, in patients with unknown COVID-19 status, did you put on...Tick all that apply

DURING THE EMERGENCY FULL LOCKDOWN

Tick all that apply.

- [ ] A surgical mask
- [ ] A FFP2/FFP3/N95 respirator mask without a valve
- [ ] A FFP2/FFP3/N95 respirator mask with valve
- [ ] Goggles and/or a shield or visor
- [ ] A second pair gloves
- [ ] A surgical cap
- [ ] Disposable shoe coverings
- [ ] N/A

49. During aerosol generating dental procedures (AGPs), in patients with unknown COVID-19 status, did you put on...Tick all that apply

DURING THE EMERGENCY FULL LOCKDOWN

Tick all that apply.

- [ ] A surgical mask
- [ ] A FFP2/FFP3/N95 respirator mask without a valve
- [ ] A FFP2/FFP3/N95 respirator mask with valve
- [ ] Goggles and/or a shield or visor
- [ ] A second pair gloves
- [ ] A surgical cap
- [ ] Disposable shoe coverings
- [ ] I did not undertake any aerosol generating procedure
- [ ] N/A

50. Did all the clinical dental team members use similar levels of protective equipment? *

DURING THE EMERGENCY FULL LOCKDOWN

Mark only one oval.

- ( ) Yes
- ( ) No
- ( ) N/A

51. Did you have problems acquiring adequate personal protective equipment? *

DURING THE EMERGENCY FULL LOCKDOWN

Mark only one oval.

- ( ) A lot
- ( ) Some
- ( ) A little
- ( ) None

52. How confident were you that you had sufficient information to have optimal infection prevention control at work? *

DURING THE EMERGENCY FULL LOCKDOWN

Mark only one oval.

- Very confident
- Confident
- Not really confident
- Not confident at all

SECTION 5. AFTER the covid-19 pandemic

53. What digital services did you use to communicate with patients?. Tick all that apply *

LAST WEEK

Tick all that apply.

- [ ] Use of personal telephone
- [ ] Use of email
- [ ] Use of social messaging (Facebook, WhatsApp, Discord...)
- [ ] Use of non-dedicated videoconferencing (Skype, Zoom, Facetime...)
- [ ] Use of a dedicated teleconsultation system
- [ ] None of the above

54. What type of dentistry did you usually provide?. Tick all that apply *

LAST WEEK

Tick all that apply.

- [ ] Special Care Dentistry
- [ ] General dentistry
- [ ] Paediatric dentistry
- [ ] Oral surgery
- [ ] Implantology
- [ ] Endodontics
- [ ] Periodontics
- [ ] Prosthodontics
- [ ] Orthodontics
- [ ] Oral Medicine

Other: [ ] ______________________

55. Did you usually have regular access to services under...Tick all that apply *

LAST WEEK

Tick all that apply.

- [ ] Oral conscious sedation (e.g. diazepam)
- [ ] Relative analgesia/nitrous oxide conscious sedation
- [ ] Intravenous conscious sedation (e.g. midazolam)
- [ ] Intravenous deep sedation (e.g. propofol)
- [ ] General anaesthesia
- [ ] None of the above

56. Your patients with special needs were mostly... *

LAST WEEK

Mark only one oval.

- ( ) Children
- ( ) Adults
- ( ) Elderly
- ( ) All ages

57. What categories of patients with special needs (principal diagnosis) did you usually provide care for? Tick all that apply *

LAST WEEK

Tick all that apply.

- [ ] Physical disability
- [ ] Intellectual / Developmental disability
- [ ] Neurological disorders
- [ ] Sensory disability
- [ ] Medically compromised patients
- [ ] Frail older adults
- [ ] Mental Health / Socially excluded
- [ ] Anxious / Phobic patients
- [ ] Other: ____________________

58. How many patients with special needs did you usually see in a week? *

LAST WEEK

Mark only one oval.

- ( ) None
- ( ) 1-10 patients
- ( ) 11-20 patients
- ( ) 21-30 patients
- ( ) 31-40 patients
- ( ) > 40 patients

59. Did you offer domiciliary dental care? *

LAST WEEK

Mark only one oval.

- ( ) Yes
- ( ) No

60. Did you find that patients were reluctant to attend for dental treatment due to fear of infection? *

LAST WEEK

Mark only one oval.

- Yes
- No

61. Did you find that dental staff were reluctant to participate in the provision of dental treatment due to fear of infection? *

LAST WEEK

Mark only one oval.

- Yes
- No

62. During oral exam and NON-aerosol generating dental procedures, in patients with unknown COVID-19 status, did you put on...Tick all that apply *

LAST WEEK

Tick all that apply.

- [ ] A surgical mask
- [ ] A FFP2/FFP3/N95 respirator mask without a valve
- [ ] A FFP2/FFP3/N95 respirator mask with valve
- [ ] Goggles and/or a shield or visor
- [ ] A second pair gloves
- [ ] A surgical cap
- [ ] Disposable shoe coverings
- [ ] N/A

63. During aerosol generating dental procedures (AGPs), in patients with unknown COVID-19 status, did you put on...Tick all that apply *

LAST WEEK

Tick all that apply.

- [ ] A surgical mask
- [ ] A FFP2/FFP3/N95 respirator mask without a valve
- [ ] A FFP2/FFP3/N95 respirator mask with valve
- [ ] Goggles and/or a shield or visor
- [ ] A second pair gloves
- [ ] A surgical cap
- [ ] Disposable shoe coverings
- [ ] I did not undertake any aerosol generating procedure
- [ ] N/A

64. Did all the clinical dental team members use similar levels of protective equipment? *

LAST WEEK

Mark only one oval.

- ( ) Yes
- ( ) No
- ( ) N/A

65. Did you have problems acquiring adequate personal protective equipment? *

LAST WEEK

Mark only one oval.

- ( ) A lot
- ( ) Some
- ( ) A little
- ( ) None

66. How confident were you that you had sufficient information to have optimal infection prevention control at work? *

LAST WEEK

Mark only one oval.

- Very confident
- Confident
- Not really confident
- Not confident at all

SECTION 6. THE SCENARIO AFTER THE COVID-19 PANDEMIC

67. In six months time, I expect that the COVID-19 pandemic will have had... *

Mark only one oval.

- A negative impact on the dental health of special care patients
- No impact on the dental health of special care patients
- A positive impact on the dental health of special care patients

68. Please explain the main reason why you think this

____

69. In six months time, I expect Universal Cross Infection Control measures in the dental surgery to... *

Mark only one oval.

- Be stricter than before the pandemic
- Return to pre-pandemic norms
- Be looser than before the pandemic

70. In six months time, I expect that special care dentistry... *

Mark only one oval.

- Will be more difficult to provide than before the pandemic
- Will have returned to pre-pandemic levels of provision
- Will be less difficult to access than before the pandemic

71. Please explain the main reason why you think this

____

This content is neither created nor endorsed by Google.

Google Forms
